# Leveraging microbiome-based interventions to improve the management of neurodegenerative diseases: evidence for effects along the microbiota-gut-brain axis

**DOI:** 10.3389/fnut.2025.1699884

**Published:** 2025-12-11

**Authors:** Noémie Auclair-Ouellet, Ola Kassem, Stéphane Bronner, Marie-Laure Oula, Sylvie Binda

**Affiliations:** Rosell Institute for Microbiome and Probiotics, Montréal, QC, Canada

**Keywords:** microbiome-based interventions, probiotics, prebiotics, synbiotics, postbiotics, next-generation probiotics, microbiota-gut-brain axis (MGBA), neurodegenerative diseases (NDDs)

## Abstract

The microbiota-gut-brain axis (MGBA) has recently emerged as a useful model for the understanding of the onset and progression of neurodegenerative diseases (NDDs). Microbiome-based interventions using biotic supplements (probiotics, prebiotics, synbiotics, postbiotics) can modulate the MGBA and constitute relevant solutions to help reduce the risk of neurological changes associated with NDDs and manage symptoms. This narrative review provides a summary of the functioning of the MGBA and of its interactions with disease processes involved in the onset and progression of NDDs. Microbiome-based interventions and their mechanisms of action are reviewed, and important considerations for the design of interventions are discussed. Next, preclinical and clinical studies on the potential of microbiome-based interventions in Alzheimer’s disease (AD), Parkinson’s disease (PD), Multiple Sclerosis (MS), Amyotrophic Lateral Sclerosis (ALS), and Huntington’s disease (HD) are reviewed. Evidence related to biomarkers of pathology (e.g., beta-amyloid or alpha-synuclein protein depositions), neuroinflammation, and metabolic activity is summarized, along with emerging evidence for the improvement of clinical symptoms and disease trajectories. Overall, preclinical studies show that microbiome-based supplements have significant positive effects on mechanisms and pathways involved in the pathophysiology of NDDs. Clinical studies show that these interventions provide important benefits both in terms of biomarkers and clinical symptoms. However, evidence is limited in some key clinical areas, such as mental wellbeing in AD and cognition in PD, and for the management of clinical symptoms in ALS and HD overall. Gaps in knowledge and open questions as well as perspectives for future research are discussed.

## 1 Introduction

According to the World Health Organization, more than 55 million people currently live with dementia, and this number is expected to reach 139 million by the year 2050 (1, 2). Dementia is a syndrome caused by a variety of diseases that damage the brain, leading to deterioration in cognitive function (e.g., memory, attention, planning, calculation, language) beyond what might be expected in normal aging. It is often accompanied by changes in mood, emotional control, behavior, or motivation (3). Dementia is already among the leading causes of mortality worldwide and is projected to be the third leading cause of death by 2040, being only surpassed by ischemic heart disease and stroke (1, 2). Despite considerable progress accomplished in the understanding of dementia and neurodegenerative diseases (NDDs) over the past 25 years, many clinical trials testing conventional medical interventions to treat those conditions have been terminated due to lack of effectiveness or adverse events (4–7). Furthermore, recently approved disease-modifying treatments have failed to deliver expected benefits (6) and other available pharmacological treatments have been shown to have limited efficacy, lose their efficacy over time, or have considerable side-effects (8–11).

Recent developments in research on the microbiota-gut-brain axis (MGBA) open new opportunities to develop microbiome-based interventions with benefits for NDD symptoms and pathophysiology (12–14). Thanks to progress in deciphering the numerous and complex interactions between the gut, its microbiota, and the nervous system, it is now possible to develop and further optimize interventions that modulate the composition of the gut microbiota to achieve positive effects that protect or even restore brain function. In humans, the gut is the body ecosystem with the largest number of microorganism species, and the largest quantity of microorganisms overall, including bacteria, fungi, viruses, and others (15, 16). Modern molecular methods have led to the identification of over 3,000 bacterial species in the gut (17). The vast majority of these bacteria belong to a small number of phyla among which Firmicutes and Bacteroidetes are dominant, followed by Actinobacteria, Proteobacteria, Fusobacteria, and Verrucomicrobia (18). While the term microbiota describes microorganisms present in a certain ecosystem, the term microbiome is broader and encompasses microorganisms, the ensemble of their genomes, as well as their structural elements, metabolites, and the environmental conditions of the ecosystem (18, 19). The gut is a complex and dynamic ecosystem that can vary due to a range of individual and environmental factors (18). Its stability, resilience, and functions can be supported by modulating the composition of the gut microbiota (16, 20). The growing evidence base for the efficacy and safety of microbiome-based supplement interventions (e.g., interventions using probiotic supplements), along with deeper insights into their mechanisms of action allow researchers, healthcare professionals, and industry members to develop solutions that exert substantial health benefits.

This narrative review provides an overview of the current understanding of the role of the MGBA in the onset and progression of NDDs and summarizes evidence on microbiome-based interventions that could prevent or delay their course and help manage core clinical symptoms. Current knowledge about those interventions’ mechanisms of action and important considerations for both preclinical and clinical studies are reviewed. Studies investigating the effects of microbiome-based solutions in Alzheimer’s disease (AD), Parkinson’s disease (PD), amyotrophic lateral sclerosis (ALS), multiple sclerosis (MS), and Huntington’s disease (HD) are reviewed, including both preclinical and clinical studies. Gaps in knowledge and perspectives for future research are presented to guide the next steps in this evolving field.

## 2 The microbiota-gut-brain axis

**FIGURE 1:**
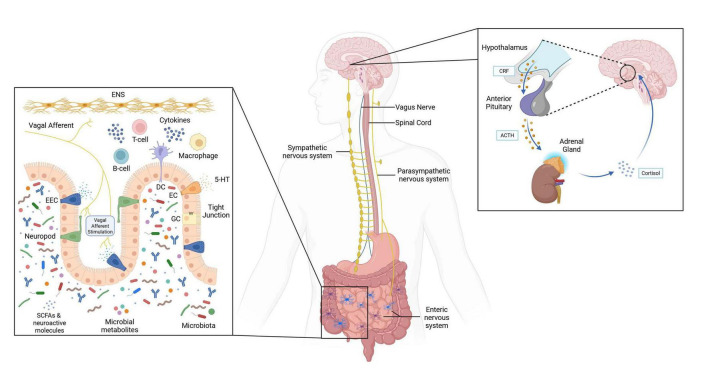
Schematic representation of the microbiota-gut-brain axis (MGBA). The immune pathway involves the interaction of the gut microbiota with immune cells, which modulates intestinal permeability and inflammation. The endocrine pathway includes the secretion of hormones by enteroendocrine cells, and the activation of the hypothalamic-pituitary-adrenal (HPA) axis during stress. The neural pathway is subtended by vagal afferent and efferent projections. The neurochemical pathway encompasses the activity of neuroactive compounds, like neurotransmitters and their precursors, secondary bile acids, and other bacterial metabolites. Created with BioRender. 5-HT, 5-hydroxytryptamine (serotonin); ACTH, adrenocorticotropic hormone; CRF, corticotrophin-releasing factor; DC, dendritic cell; EC, enterochromaffin cell; EEC, enteroendocrine cell; ENS, enteric nervous system; GC, goblet cell; SCFA, short-chain fatty acids. Created with BioRender.com.

The microbiota-gut-brain axis (MGBA) is defined as the ensemble of bidirectional interactions between the gut, its microbiota, and the brain (12–14). Signals from the brain can influence sensorimotor and secretory functions of the gut, and reciprocally, the gut can produce signals that modulate brain functions. The numerous and complex interactions that are subtended by the MGBA involve the enteral, peripheral, and central nervous system (CNS), and are mediated by immune, endocrine, neural, and neurochemical pathways. Communications along these various pathways are thought to be mediated by metabolites produced by gut bacteria, including neurotransmitters and their precursors, short-chain fatty acids (SCFA; mainly acetate, butyrate, and propionate), and secondary bile acids (14, 21, 22). A schematic representation of the MGBA is provided in Figure 1.

Through the immune pathway, gut bacteria metabolites interact with intestinal epithelial cells to reduce gut permeability and enhance barrier integrity by strengthening tight junctions and increasing transepithelial electrical resistance (TEER). Metabolites also interface with the immune system and modulate the production of cytokines that regulate inflammatory responses, and thereby influence systemic inflammation, which has been implicated in NDDs and several other conditions (23–25). In recent years, more attention has been drawn to the role of microbiota-released extracellular vesicles (MEVs) in the transmission of immune signals from bacteria to host cells (26, 27). MEVs are a specific type of extracellular vesicles (EVs), which is a collective term that describes “particles naturally released from the cell that are delimited by a lipid bilayer and cannot replicate, i.e., do not contain a functional nucleus” (28). Particles contained in MEVs include proteins, lipids, nucleic acids, and metabolites. Their role would encompass both interactions between bacteria (e.g., biofilm formation, horizontal gene transfer) and host-bacteria interactions (e.g., toxin delivery, pathogenicity). MEVs can contain antigens that activate Toll-like receptors on epithelial or immune cells and increase blood-brain-barrier (BBB) permeability, thus facilitating the entry of bacterial byproducts in the brain (26, 29). In addition, SCFAs can cross the BBB and reach the brain, but their uptake appears to be limited. SCFAs are thought to promote BBB integrity by inhibiting inflammatory pathways. Gut bacteria metabolites also affect microglia structural and functional integrity, thereby influencing brain immune response and neuroinflammation (22). Neuroinflammation describes inflammatory responses within the brain and spinal cord (30–32). It is mediated by cytokines, chemokines, reactive oxygen species, and secondary messengers produced by microglia and astrocytes, endothelial cells, and immune cells in response to infections, injuries, or diseases. Similar to other inflammatory responses, neuroinflammation can be adaptive, for example in the context of immune conditioning (31). However, when it is dysregulated and becomes chronic, negative effects emerge (30–32). There is growing evidence for the role of immune-related neuroinflammation in the development of neurodegeneration, with evidence tying it to the disease process of several NDDs (30–32).

The endocrine pathway involves interactions between metabolites and their receptors, which can induce the secretion of hormones from enteroendocrine L cells and lead to indirect signaling to the brain through systemic circulation or vagal pathways. In healthy functioning, these mechanisms play an important role in the regulation of appetite and food intake. Other hormones influenced by gut bacteria metabolites include leptin, ghrelin and insulin. Ghrelin is associated with food-related rewards and enjoyment. It also modulates stress and psychological responses through the hypothalamic-pituitary-adrenal (HPA) axis and other neural pathways. Altered insulin metabolism is a risk factor for metabolic and cardiovascular conditions and has also been implicated in the pathophysiology of AD and PD (33, 34). The HPA axis is a major neuroendocrine system that is responsible for the response to stress and the regulation of body processes, including digestion and immune responses. During stress, the hypothalamus secretes corticotrophin-releasing factor (CRF), which triggers the release of adrenocorticotropic hormone (ACTH) by the pituitary gland, and in turn, the release of glucocorticoids (e.g., cortisol) by adrenal glands. CRF activates receptors located in the CNS and gastrointestinal (GI) tract, and induces various effects on GI function, such as delaying gastric emptying, altering GI motility, and increasing intestinal permeability (35).

Although vagal afferents do not directly interface with the gut lumen or microbiota, they can be activated by microbial metabolites, thereby transmitting signals to the brain. Cells in contact with the gut lumen can also interface with the vagus nerve and trigger signals to the CNS. The vagus nerve is composed of 80% of afferent fibers and 20% of efferent fibers. Its afferent fibers relay interoceptive signals to the brain through mechano-, chemo-, thermo- and osmo-receptors from various organs, including the gastrointestinal tract, with substantial effects on emotions and psychological well-being (36–38). For instance, alterations in vagal signals increase anxiety and depressive symptoms (38). Conversely, regulation of vagal signals through vagus nerve stimulation (VNS) improves mood in individuals with treatment-resistant depression and decreases anxiety- and depression-like behaviors in rodents (38).

Through their metabolic activity, gut bacteria play an important role in the production of molecules involved in neurochemical signaling, including neurotransmitters and their precursors, and secondary bile acids (14, 21, 39). Bacteria may play a direct role in the production of those molecules or perform metabolic activities that increase the bioavailability of intermediate compounds, which can be used by other bacteria to synthesize neurotransmitters and other metabolites. This mechanism, cross-feeding, is also involved in the production of SCFAs (40, 41). The synthesis of serotonin (5-HT) from tryptophan is a well-known example of the role of gut bacteria in the synthesis of neurotransmitters. More than 90% of serotonin is produced by enterochromaffin cells in the gut, the rest being synthesized in the brain (22). Gut bacteria metabolites would influence the production of serotonin in multiple ways, including by promoting the expression of tryptophan hydroxylase genes (22) and downregulating the activity of the kynurenine pathway, thus increasing the bioavailability of tryptophan for serotonin synthesis (42). Beyond serotonin, gut bacteria, especially from the *Bifidobacteriaceae*, *Bacillaceae*, *Lactobacillaceae*, and *Streptococcaceae* families, have been involved in the synthesis of glutamate, gamma-aminobutyric acid (GABA), norepinephrine (noradrenaline), and histamine, to name a few (21, 39). Gut bacteria also modulate levels of brain derived neurotrophic factor (BDNF), which is involved in synaptic formation, plasticity, neuroimmune response, and neural survival (39).

### 2.1 The MGBA in the onset and progression of NDDs

Gut dysbiosis, which can be defined as an imbalance in the composition and relative abundance of bacterial taxa normally present in the gut, has been observed in several NDDs (43–45) (further reviewed in section “4 Effects of microbiome-based interventions in NDDs”). Gut dysbiosis is associated with instability in the composition of the gut microbiota, a reduction in the abundance of beneficial taxa, a concomitant increase in the abundance of opportunistic bacteria and pathogens, and overall changes in microbial diversity (20, 46). As a result, immune and metabolic functions normally performed by beneficial bacteria are impaired, detrimental effects associated with the presence of opportunistic bacteria and pathogens are increased, and the host becomes more vulnerable to diseases and infections (47).

The term “leaky gut syndrome” has been used to describe increased gastrointestinal permeability related to the loss of tight junctions and degradation of mucosal lining (25, 45, 48). The loss of gastrointestinal barrier integrity leads to the translocation of bacteria in the intestinal epithelium. In addition to triggering inflammation, the translocation of bacteria can lead to their displacement to other body sites and the proliferation of pathobionts in other body ecosystems (14, 25). Relatedly, alterations in BBB integrity and increased permeability have been associated with the pathophysiology of NDDs (4, 24, 49, 50).

The immune response triggered by gut dysbiosis and ensuing inflammation is thought to be the key factor linking alterations in the gut microbiota and NDDs (51, 52). Inflammation would be driven by various cytokines, chemokines, proteases, and growth factors. The term “inflammaging” has been coined to describe a state of chronic low-grade inflammation that is commonly found in conditions whose prevalence increases with aging (53, 54), including but not limited to NDDs: cardiovascular diseases, autoimmune diseases, and certain types of cancers. Interestingly, some of these conditions, such as cardiovascular diseases, are considered to increase risks or to be frequent comorbidities of NDDs, supporting the existence of common underlying factors.

Oxidative stress (or disrupted redox balance) is a state of imbalance between the production of reactive oxygen species (ROS) resulting from normal metabolic activity (e.g., digestion, respiration) and exposure to stressors (e.g., pollution, UV light, immune-related inflammation, pathogens, and lifestyle-related factors like alcohol consumption and smoking), and the body’s capacity to manage them (55, 56). Oxidative stress describes the downstream effects of systemic inflammation and helps explain how they are exacerbated and perpetuated. It is characterized by oxidative damage to intracellular components (proteins, lipids, DNA) causing the production of endogenous damage-associated molecular patterns (DAMPs) and the release of cytokines (52, 56). DAMPs, which are activated in response to cellular damage, can recruit immune cells that promote inflammation. Cytokines released in response to oxidative stress further contribute to systemic inflammation, reinforcing oxidative stress, and thus leading to chronic inflammation (52).

Having a poor diet has also been identified as a major contributor to the production of ROS. Diets rich in fat, sugar, processed food, and red meat (e.g., Western diet) would contribute to systemic inflammation and have profound impacts over the gut microbiota (57, 58). The spread of the Western diet globally has been accompanied by an increase in the prevalence of non-communicable diseases, including metabolic diseases (obesity, type 2 diabetes), cardiovascular diseases, gut and liver diseases, some forms of cancer, and NDDs (57, 58). Notably, certain gut bacteria metabolize L-carnitine and other molecules contained in red meat and other animal products to produce trimethylamine (TMA), which is further metabolized into trimethylamine N-oxide (TMAO) through oxidation (59, 60). Elevated plasma concentrations of TMAO are associated with metabolic and cardiovascular risk factors and diseases, and with cerebrovascular diseases (e.g., stroke) (61, 62). Cerebrovascular diseases have been associated with cognitive decline and dementia (c.f., vascular dementia (63)), and represent a common comorbidity with significant impacts on the onset and progression of NDDs (4, 64, 65).

Neurodegenerative diseases (NDDs) are related with the presence of abnormal protein folding, aggregation and deposition in the brain, which lead to alterations in neural functioning, and ultimately, neural cell death (66). For instance, beta-amyloid aggregates are among the leading factors of AD pathology. *Escherichia coli* and other gut bacteria produce “curli,” a type of extracellular fibers that share properties with amyloids (66). In health, bacterial amyloids contribute to bacterial cell binding and formation of biofilm, increasing resistance to physical and immunological challenges (66, 67). The role of bacterial amyloid in NDD pathology has not been elucidated, but some hypotheses have been proposed. Dysbiosis could lead to excess production of bacterial amyloid, or alterations to amyloid structure, which could trigger the immune system and generate inflammation. Bacterial amyloid could spread to the brain in a prion-like fashion and contribute to aggregate formation, in addition to triggering further amyloid production by neuronal proteins (66).

Commensal bacteria of the gastrointestinal tract, including taxa from the *Lactobacillus*, *Bifidobacterium*, *Bacteroides*, *Faecalibacterium*, *Roseburia*, *Prevotella*, and *Ruminococcus* genus, perform metabolic activities that lead to the production of compounds with essential roles in the mediation of communication along the MGBA, such as SCFA, secondary bile acids, and neurotransmitters and their precursors (14, 21, 22). Other roles played by gut bacteria metabolic outputs include promotion of nutrient uptake in the large intestine, lipogenesis, and regulation of apoptosis. Gut dysbiosis leads to a loss of bacteria able to perform those key metabolic functions directly, or to contribute to the production of intermediate compounds further metabolized by other bacteria through cross-feeding (40, 41). In addition to the loss of beneficial metabolic activity related to reduced abundance and diversity of beneficial taxa, recent studies point to excess abundance of bacteria with a specific metabolic activity as a potential disease-causing mechanism. Notably, many studies highlight the role of bacteria involved in the metabolism of sulfur as contributors to pathophysiology, especially *Desulfovibrio* spp. (68–70). At normal concentrations, hydrogen sulfide resulting from sulfur metabolism is cytoprotective, maintaining the integrity of the mucus layer. When it is present in excess, it causes dysbiosis and reduces the abundance of butyrate producers, and increases inflammation, oxidative stress, and mitochondrial damage. One study found that *Desulfovibrio* induced alpha-synuclein aggregation in a *Caenorhabditis elegans* model of PD (71). Increased abundance of *Desulfovibrio* has been found in humans with PD (68, 69, 72), and MS (73). Furthermore, alterations in sulfur metabolism have been reported in AD and PD (70). In summary, gut dysbiosis would be related to the onset and progression of NDDs in several ways. Local and systemic inflammation associated with dysbiosis appears to be at the center of the cascade of detrimental changes that impair body functioning and ultimately lead to clinical manifestations.

## 3 Microbiome-based interventions

The numerous interactions between the microbiota, the gut, and the brain, and findings highlighting how these interactions are disrupted in NDDs motivate the development and optimization of interventions to modulate them. A brief description of common microbiome-based interventions is provided to set the stage for the review of findings specific to different NDDs in the following sections.

### 3.1 Types of microbiome-based interventions

Common microbiome-based interventions rely on supplementation to provide the gut microbiome with beneficial microorganisms and nutrients that can support its functions and achieve beneficial effects on host health. In the past 10 to 15 years, new types of interventions have emerged. While they are promising, more studies are needed to establish the feasibility of these interventions, as well as their safety and efficacy. In addition to interventions relying on supplements, other types of approaches that can modulate the composition of the gut microbiota are briefly presented.

#### 3.1.1 Probiotics, prebiotics, and synbiotics

Probiotics represent the best known and most studied type of microbiome-based supplement. They are defined as “live microorganisms that, when administered in adequate amounts, confer a health benefit on the host” (74). Most probiotics are bacteria, but some yeasts (e.g., *Saccharomyces cerevisiae* var. *boulardii*) have been shown to have probiotic properties. The most studied probiotic bacteria are from the Bifidobacteriaceae (e.g., *Bifidobacterium bifidum*, *Bifidobacterium longum*) and the Lactobacillaceae (e.g., *Lacticaseibacillus rhamnosus*, *Lactobacillus helveticus*) families. Prebiotics are defined as “a substrate that is selectively utilized by host microorganisms conferring a health benefit” (75). They include fermentable dietary fibers, oligosaccharides, polyunsaturated fatty acids (PUFA), conjugated linoleic acids (CLA), and other nutrients. By providing substrate for fermentation to bacteria, prebiotics support their metabolic activity and can thus influence microbiota composition by promoting the growth of bacteria that use specific substrates. Synbiotics are “a mixture comprising live microorganisms and substrate(s) selectively utilized by host microorganisms that confers a health benefit on the host” (76). Synbiotics can be composed of a prebiotic and a probiotic administered together, each achieving positive effects on host health. Alternatively, synbiotics can be composed of live microorganisms and substrates that may or may not have proven probiotic and prebiotic properties, respectively, and that exert synergistic effects with positive impacts on host health. For example, a synergistic synbiotic may involve the combination of a bacteria known to metabolize a specific dietary fiber to increase levels of a specific metabolite that exerts positive effects on host health (76).

#### 3.1.2 Postbiotics

Postbiotics refer to a “preparation of inanimate microorganisms and/or their components that confers a health benefit on the host” (77, 78). According to this definition, postbiotics do not solely refer to microorganisms’ metabolic outputs, although they can be part of postbiotic preparations. The focus is on the inanimate nature of microorganisms used as postbiotics. Although debate remains regarding the nature, definition, and methodology surrounding the development of postbiotics, they offer therapeutic potential and some specific advantages, such as their ability to resist harsh gastrointestinal conditions, their longer shelf life and the lack of refrigeration requirements. However, challenges remain regarding variability in functionality due to inactivation methods, and the need for more robust clinical evidence to support their use in routine practice (79).

#### 3.1.3 Next-generation probiotics

With significant advancements in next-generation sequencing (NGS) and bioinformatic platforms, the concept of next-generation probiotics (NGPs) has emerged. NGPs are defined as microbes that are not traditionally used as probiotics and whose health benefits have been identified through microbiome-wide association studies in health and disease cohorts (80, 81). NGPs tend to be strict anaerobes, often from taxa considered difficult to culture, such as *Faecalibacterium prausnitzii*, *Akkermansia muciniphila*, *Eubacterium hallii*, *Bacteroides fragilis*, and *Christensenella minuta*. These bacteria can be used as markers of a healthy microbiome, which is supported by their ability to produce metabolites with immunomodulatory, metabolic, and neuroactive properties relevant to the host health (80, 81). A prime candidate among NGPs is *Akkermansia muciniphila*, a Gram-negative, mucin-degrading bacterium first isolated from human feces in 2004 (82). It constitutes 0.5%–5% of the total gut bacterial population and is thought to play a central role in gut barrier maintenance, mucosal immunity, and metabolic regulation (83). Additionally, *A. muciniphila* produces extracellular vesicles, which serve as carriers of microbial metabolites, proteins, and RNA (83). While studies have shown the benefits of *A. muciniphila* on metabolic and inflammatory diseases, its increased abundance has also been reported in neurodegenerative diseases such as MS and PD, indicating context-dependent effects and highlighting the need for disease-specific functional studies (73, 80–82).

#### 3.1.4 Engineered probiotics

Engineered probiotics represent a new avenue in microbiome-based interventions that combines the use of traditional probiotics with modern gene-editing technologies to create interventions that aim to enhance safety and specificity (84). Engineered strains of *E. coli* Nissle 1917 and *Lactobacillus* spp. have been developed to target a range of diseases, including inflammatory bowel disease (IBD), metabolic disorders, and cancer (84). For example, engineered *E. coli* Nissle 1917 was used to locally deliver immune checkpoint (PD-L1 and CTLA-4), which resulted in tumor regression in mouse models, while minimizing toxicity typically associated with antibody therapies (85). The development of engineered probiotics is still in its early days, and numerous challenges related to regulatory acceptance, safety of genetically modified organisms, and the limited availability of editable probiotics still need to be addressed (84). Further studies are required to determine their applicability in the context of NDD interventions.

#### 3.1.5 Microbiota-released extracellular vesicles

The use of MEVs is another novel potential strategy aiming to improve safety and treatment precision. The use of MEVs would allow targeted delivery of bioactive molecules that mirror the benefits of their originating microbe (86). Internally produced MEVs have been suggested to mediate communication along the gut-brain axis and contribute to the pathogenesis of neurodegenerative disorders like AD and PD. In the same logic, supplying external MEVs from probiotic strains could potentially confer neuroprotective and immunomodulatory effects, especially owing to their ability to cross gut and blood-brain barriers (87). While evidence remains limited, some studies have suggested a role in regulating microglial activity, reducing neuroinflammation, modulating neurotransmitter levels (e.g., serotonin), and influencing neuronal signaling. For example, MEVs from *Lactobacillus plantarum* have been shown to exert antidepressant effects in male C57BL/6J mice (88). While this technology is promising, challenges remain regarding the production and distribution of MEVs, in addition to their regulation. Furthermore, more studies are needed to establish their safety and efficacy.

#### 3.1.6 Other interventions

Other types of interventions can be used to modulate the gut microbiota, such as diets (e.g., Mediterranean diet), fecal matter transplant, and antibiotics (57, 89, 90). Dietary approaches, such as the Mediterranean diet, exert beneficial effects through multiple mechanisms (91). This diet consists of a plant-forward eating pattern rich in fruits, vegetables, whole grains, legumes, and healthy fats, known for promoting metabolic health. A systematic review and meta-analysis of 37 observational and intervention studies reported that adherence to a Mediterranean-style diet significantly enriched microbial diversity, and particularly increased the abundance of SCFA-producing taxa such as *Faecalibacterium*, *Roseburia*, and *Prevotella*, leading to improvements in bowel health, glycemic control, lipid profiles, and inflammation markers (91). With respect to NDDs, a large cohort study found that each incremental point in Mediterranean diet adherence was associated with a 37% reduction in MS risk, partially mediated by gut microbial alterations (92). However, another systematic review including 17 observational studies and 17 RCTs failed to find evidence of a consistent association between the Mediterranean diet and gut microbiota metabolism and composition (93). Aside from yielding mixed results, diets may be difficult to implement due to higher costs, food access challenges, cultural dietary preferences, and compliance issues further compounded by modern lifestyle factors such as time constraints and irregular schedules (57, 94).

Fecal matter transplant (FMT) is a procedure involving transplanting minimally processed stool from a healthy donor to a patient with the purpose of restoring microbial balance and exerting a therapeutic effect (89). FMT has been shown to help treat *C. difficile* infection, IBD, psychological symptoms of irritable bowel syndrome (IBS), and metabolic syndrome (95). In NDDs, preclinical studies have shown that FMT can reduce amyloid-beta plaque accumulation, tau phosphorylation, and neuroinflammation, while improving cognitive function and gut barrier integrity (96). Nevertheless, FMT is limited by the need to identify suitable donors, risks associated with the unintended transfer of pathogenic microorganisms, variable protocols and lack of procedure standardization, in addition to the risks associated with delivery methods (colonoscopy) (96, 97).

Lastly, while antibiotics can improve NDD-related outcomes (e.g., reduce neuroinflammation, reduce amyloid and tau depositions, regulate cholinergic transmission), they have substantial side effects (e.g., antibiotics-associated diarrhea) and lead to gut dysbiosis, which question their use, especially as a long-term intervention, for the management of gut microbiota changes in NDDs (90).

### 3.2 Mechanisms of action

The human gut is home to over 3,000 bacterial species, grouped in six predominant phyla. While having a consensus definition of a healthy gut microbiome is desirable from a research perspective, it is a very difficult goal to achieve due to individual variability and the influence of age, host health, diet, lifestyle, and environmental factors (18, 98, 99). However, keystone taxa associated with positive health outcomes and resilience in the face of challenges and aggressions are starting to emerge, thanks to large scale studies involving the analysis of several thousands of gut microbiota samples collected worldwide (98). Although these results require replication and validation, they constitute an important step toward a better understanding of the interplay between the gut microbiome and host health. Through their ability to influence the composition of the gut microbiota by exerting beneficial metabolic activity, cooperation with commensal bacteria, and competition with pathogens, microbiome-based solutions would help maintain or restore gut homeostasis (16, 20, 100).

Bacteria can cooperate with others through cross-feeding, a processus by which some taxa use metabolic outputs of other taxa to synthesize different substances (41). As explained previously, bacteria’s metabolic outputs, and especially SCFAs (mainly acetate, butyrate, and propionate) play several key roles in the maintenance of gastrointestinal health, and in the mediation of communication along the MGBA (22, 41). The balance of SCFA producers and supporting bacteria, as well as sufficient supply of fermentation substrate, would ensure adequate amounts of these compounds. By maintaining and restoring gut homeostasis, microbiome-based interventions could also reduce or stop excessive or abnormal production of bacterial amyloid, thus potentially managing bacterial-induced protein depositions and aggregates (66). Similarly, this could regulate the production of metabolites that have detrimental effects when they are present in excess (70).

Bacteria also cooperate in the formation of biofilm, which can be defined as “aggregates of microorganisms in which cells are frequently embedded in a self-produced matrix of extracellular polymeric substances (EPS) that are adherent to each other and/or a surface” (101). Biofilm has emergent properties and provides several benefits to communities of bacteria, including shelter against dehydration, capture of nutrients, and protection against stressors (100, 102). The net impact of biofilm can be positive or negative for the host depending on composing bacteria and their interactions (16).

On the other hand, bacteria can compete for adhesion on the surface or epithelial cells and for nutrients (20, 100, 102). Likewise, some probiotic bacteria can disrupt the formation of biofilms by pathobionts and antagonize these taxa through the production of organic acids that modulate pH levels, and by the production of bacteriocins that reduce pathogens’ viability and pathogenicity (16, 20, 102).

Probiotics can adhere to host cells and induce immune signals that help maintain or restore gastrointestinal barrier integrity (16, 20, 102). They help strengthen tight junctions and promote the secretion of mucus, which acts as a lubricant during gastrointestinal transit but also confers protection from substances and pathobionts present in the gut lumen (20). Probiotics can have several effects on the immune response by supporting the balance of pro-inflammatory (e.g., IL-8, TNF-α) and anti-inflammatory cytokines (e.g., IL-10). Overall, this results in a reduction of oxidative stress and systemic inflammation (20, 100, 102).

Microbiome-based interventions also support the microbiota’s activity in the deconjugation of bile acids, which is essential in the metabolism of fat from nutrition and the absorption of fat-soluble vitamins (20). Bacteria in the ilium metabolize primary bile acids and turn them into secondary bile acids. This mechanism is thought to subtend probiotics’ hypocholesterolemic effects and downstream benefits on metabolic and cardiovascular risk factors. Relatedly, probiotics have been shown to regulate insulin metabolism (20).

This short summary does not cover all the ways in which microbiome-based interventions exert beneficial effects on host health but provides an overview of the scope and diversity of their mechanisms of action. Later sections of the paper that focus on the effects of microbiome-based interventions in preclinical models and individuals with NDDs will further expand on the current understanding of these interventions’ mechanisms of action.

### 3.3 Important considerations for the intervention

In recent years, increasing emphasis has been put on preventing the pathological processes that lead to NDDs, ideally long before clinical manifestations appear. This approach is coherent with call-to-actions to focus on modifiable risk factors of NDDs when designing interventions (103), and with current diagnosis guidelines that put forward the identification of biomarkers in asymptomatic individuals (65). Considering the role of gut dysbiosis in early disease pathogenesis, microbiome-based solutions could be initiated as a risk reduction measure in the presence of lifestyle-related risk factors, or upon detection of biomarkers (104).

Some estimates suggest that up to one-third of dementia cases may be avoided by addressing modifiable risk factors along the life course (105). This perspective, supported by the Lancet Commission on Dementia Prevention, Intervention and Care, is grounded in large-scale observational studies linking factors such as low educational attainment, midlife hearing loss, hypertension, obesity, smoking, depression, diabetes, physical inactivity, and social isolation to increased dementia risk (103). It is worth noting that among those risk factors, many are closely related to diet (hypertension, obesity, and diabetes), which supports the use of microbiome-based supplement interventions. Modifiable risk factors are theorized to influence neuropathological processes decades before clinical onset, highlighting the importance of early and sustained preventive strategies. However, despite theoretical promise, findings from large randomized controlled trials using lifestyle modification approaches have yielded modest or inconclusive results (106). Furthermore, the lack of clarity regarding the nature and design, optimal timing, duration, and intensity of such interventions, as well as the heterogeneity in individual risk profiles and disease trajectories, complicates their translation into actionable public health recommendations. Ethical considerations also arise when preventive strategies are applied broadly to asymptomatic individuals without robust predictive tools, as some may never develop dementia despite the presence of risk factors or biomarkers. While cerebrospinal fluid (CSF) samples remain the gold standard for beta-amyloid and phosphorylated Tau quantification, plasma-based biomarkers have gained accuracy and scalability, offering cost-effective and minimally invasive alternatives (65, 107). However, challenges remain, including variability in assay standardization, limited disease specificity for certain markers, and uncertainty around predictive thresholds and clinical interpretation, particularly in asymptomatic individuals. Future directions may emphasize composite biomarker panels, longitudinal studies for validation, and integration with neuroimaging and clinical phenotyping to improve accuracy (107). With respect to microbiome-based interventions, microbiome-informed risk profiles may eventually serve as both a screening tool and a guide for intervention selection and success. Implementing advanced “omics” such as strain-level resolution metagenomics and metabolomics in longitudinal studies could yield distinctive patterns and support the notion of microbiome signature as a biomarker (104).

The use of microbiome-based interventions in early symptomatic individuals may modulate ongoing neuroinflammatory processes and slow down or delay disease risk or progression (54, 96). In that context, microbiome-based interventions could be used to strengthen defenses that limit ongoing damage and manage symptoms. Some have even proposed that restoration of function should not be automatically discounted, considering the plasticity of the CNS (66). Meaningful outcomes would therefore include stabilization of function, reduced rate of functional decline, and improved function, which highlights the need to establish a solid baseline and define clear outcomes. Considering the heterogeneity and complexity of NDD syndromes and disease trajectories, outcomes need to be carefully selected to capture key changes with respect to their role in the disease process and impact on daily functioning and quality of life (108). On the biological level, changes in fluid biomarkers, inflammatory markers, SCFA levels, and intestinal permeability markers can offer mechanistic insights and contribute to elucidating pathophysiology (16, 20). Additionally, changes in microbiota composition, such as increased alpha diversity, higher relative abundance of beneficial genera and reduction of pro-inflammatory taxa may suggest microbiome restoration and correlate with other disease markers, even though such changes may not always correlate with clinical outcomes (109).

Whether the intervention is used as a risk reduction measure or in symptomatic individuals, long-term use of microbiome-based supplements may be necessary to maintain or restore microbial balance and exert systemic benefits. In general, optimal timing and duration for microbiome interventions may vary by disease, intervention type, population, and should be empirically determined in longitudinal trials. Alternative delivery routes for the supplements may need to be considered in advanced stages or in the presence of specific symptoms (e.g., dysphagia, gastrointestinal dysmotility).

## 4 Effects of microbiome-based interventions in NDDs

Several studies have focused on investigating the effects of microbiome-based supplement interventions in NDDs, both in preclinical animal models and in clinical trials. The next sections present the evidence available for those interventions in five NDDs: Alzheimer’s disease (AD), Parkinson’s disease (PD), Multiple Sclerosis (MS), Amyotrophic Lateral Sclerosis (ALS), and Huntington’s disease (HD). Summaries of evidence are preceded by a short description of disease symptoms, epidemiology, pathophysiology, and changes in gut microbiota. The review focuses on microbiome-based interventions using “biotics” supplementation: probiotics, prebiotics, synbiotics and postbiotics. A summary of key findings is provided in Table 1.

**TABLE 1:** Summary of main effects of microbiome-based interventions in NDDs.

NDD	Preclinical studies	Clinical studies
Alzheimer’s disease and Mild Cognitive Impairment	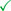 Reduction in beta-amyloid deposition 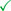 Better cognitive performance in common tests of short-term memory (Y-maze), long-term memory (passive avoidance test), and spatial recognition (Morris Water Maze) 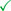 Better performance in other cognitive tests (e.g., Novel Object Recognition) 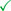 Reduction in neuroinflammation markers (e.g., TNF-α, IL-1) 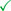 Increase in BDNF 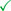 Downregulation of autophagy and apoptosis 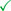 Regulation of cholinergic transmission - Increase in alpha and/or beta diversity of gut microbiota in some studies - Decrease in markers of oxidative stress in some studies 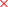 No reduction of tau hyperphosphorylation 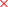 No changes in GFAP	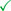 Improvement in global cognition in AD 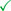 Improvement in some cognitive domains: recall/delayed memory, attention, and visuospatial/constructional abilities 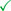 Reduction in MDA and hs-CRP 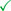 Increase in TAC 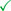 Reduction in HOMA-IR and triglyceride levels - Improvement in global cognition in MCI in some studies 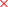 No change in some cognitive domains: orientation, registration/immediate memory, and language 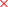 No effects on glutathione and nitric oxide 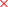 No change in QUICKI, total cholesterol levels, and VLDL
Parkinson’s disease	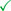 Preservation of dopaminergic cells 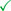 Reduction of α-synuclein aggregation 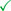 Reduction of oxidative stress 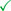 Reduction of inflammation 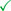 Restoration of neurotrophic pathways 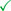 Improvement of motors symptoms 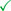 Improvement of non-motor symptoms, including cognition	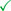 Improvement in the frequency of complete bowel movements 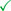 Improvement in constipation (PAC-QOL, Wexner score) 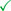 Improvement in anxiety (HAMA) 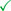 Improvement in non-motor symptoms (MD-UPDRS I, NMSS) 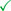 Improvement in functional abilities in ADLs (PDQ-39) 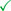 Reduction in intestinal inflammation (decreased calprotectin levels) 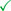 Increase in fecal levels of butyrate - Improvement in sleep quality pre-post (PDSS) - Improvement in stool consistency (BSS) in some studies - Improvement in depression in some studies (HAMD-17, BDI) - Improvement in global cognition pre-post in some studies (MMSE) - Increase in the abundance of bacteria that produce key metabolites in some studies 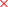 No change in defecation effort 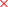 No change in motor symptoms (MDS-UPDRS III) 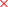 No change in bowel habits measured with the CSS
Multiple sclerosis	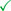 Decrease in levels of IFN-γ, IL-17, IL-6, and TNF-α 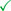 Increase in levels of IL-10 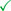 Modulation of the numbers of T-reg CD4/CD25 cells 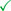 Improvement in the animal equivalent of clinical scores and the severity of symptoms 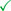 Reduction in the incidence of symptoms after EAE induction 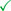 Delay in symptom onset after EAE induction 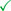 Improvement in gastrointestinal motility 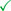 Increase in the relative abundance of SCFA producers and concentration of butyrate 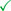 Reduction in CNS inflammation and demyelination 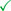 Decrease in the proportion and numbers of pathogenic Th17 cells in the brain and spleen - Reduction in the duration of disease in studies using *E. faecium* - Reduction in mortality in female animals only	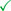 Improvement in clinical scores (EDSS) 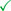 Improvement in mental health (depression, fatigue, pain) 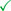 Improvement in general health 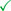 Modulation of inflammation markers: reduction of IL-6, CRP, TNF-α, IFN-γ and fecal calprotectin, and increase of IL-10, TGF-β1 and FOXP3 levels 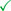 Modulation of oxidative stress markers: reduction of hs-CRP, and increase of nitric oxide levels and serum antioxidant capacity 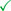 Improvement in insulin and cholesterol metabolism 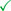 Modulation of the gut microbiota composition 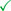 Modulation of gut-microbiota-related metabolic pathways 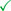 Induction of anti-inflammatory peripheral immune response on monocytes and dendritic cells 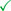 In controls: reduction in the expression of MS risk allele HLA-DQA1 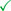 Improvement in bladder and bowel control 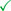 Improvement in sexual function - Improvement in some subscales of a quality-of-life tool (36-Item Short Form Survey)
Amyotrophic lateral sclerosis	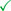 Reduction in inflammatory markers (IFN-γ) 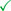 Reduction in motor symptoms and paralysis 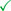 Restoration of lipid metabolism homeostasis and energy balance 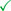 Delay in disease onset and progression 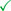 Reduction of motor neuron loss 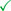 Improvement in mitochondrial function in skeletal muscles and reduction of atrophy 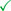 Reduction in the activation of astrocytes and microglia 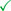 Modulation of the gut microbiota 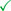 Increase in SCFA levels 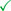 Enhanced autophagy flux 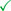 Reduction in SOD1 aggregation 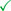 Improvement in gene expression patterns in the spinal cord 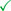 Enhanced intestinal motility 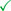 Decrease in intestinal permeability	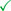 Modulation of the gut microbiota composition 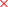 No effect on the progression of the disease (ALSFRS-R)
Huntington’s diseases	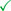 Reduction of paralysis 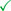 Improvement in lipid metabolism	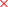 No effect on gut dysbiosis and gastrointestinal symptoms 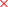 No effect on cognition 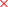 No effect on mood

AD, Alzheimer’s disease; ADLs, activities of daily living; ALSFRS-R, Amyotrophic Lateral Sclerosis Functional Rating Scale–Revised; BDI, Beck Depression Inventory; BDNF, brain-derived neurotrophic factor; BSS, Bristol Stool Scale; CNS, central nervous system; CRP, C-reactive protein; CSS, Constipation Scoring System; EAE, experimental autoimmune encephalomyelitis; EDSS, Expanded Disability Status Scale; FOXP3, forkhead box P3 gene; GFAP, glial fibrillary protein; HAMA, Hamilton Anxiety Scale; HAMD-17, Hamilton Depression Scale-17; HLA-DQA1, Major histocompatibility complex, class II, DQ alpha 1 gene; HOMA-IR, homeostatic model assessment for insulin resistance; hs-CRP, high-sensitivity C-reactive protein; IFN-γ, interferon gamma; IL, interleukin; MCI, Mild Cognitive Impairment; MDA, malondialdehyde; MDS-UPDRS, Movement Disorder Society Unified Parkinson’s Disease Rating Scale; MMSE, Mini Mental State Examination; MoCA, Montreal Cognitive Assessment; MS, Multiple Sclerosis; NMSS, Non-Motor Symptoms Scale; PAC-QOL, Patient Assessment of Constipation Quality of Life Questionnaire; PD, Parkinson’s disease; PDQ-39, Parkinson’s Disease Questionnaire, 39-item version; PDSS, Parkinson’s Disease Sleep Scale; QUICKI, quantitative insulin sensitivity check index; SCFA, short-chain fatty acids; SOD1, superoxide dismutase 1 gene; TAC, total antioxidant capacity; TGF-β1, transforming growth factor-beta 1; Th17, T helper 17; TNF, tumor necrosis factor; T-reg, regulatory T cells; VLDL, very-low-density lipoprotein.

While this review uses a narrative approach, it aims to present a comprehensive summary of studies investigating the effects of microbiome-based interventions in NDDs. To ensure adequate coverage of the literature, studies have been identified through a search on PubMed using the name of each NDD as a MeSH term combined with each biotic intervention in the Title/Abstract field. Systematic reviews and meta-analysis were reviewed when they were available and when they included more than 10 original studies. Case reports and small sample observational studies that lacked control conditions or groups were excluded. The initial search was performed on June 5, 2025, and updated on July 30, 2025. A search through the reference list of selected articles and on Google Scholar was also performed.

### 4.1 Alzheimer’s disease

Alzheimer’s disease (AD) is associated with the accumulation of misfolded protein aggregates (beta-amyloid plaques and tau neurofibrillary tangles) that lead to loss of neuron function and neuronal death (65). Clinically, AD is typically manifested by episodic (long-term) memory impairments, which progressively extend to other domains of cognition, reasoning, and behavior. As the disease progresses, cognitive impairments affect functional abilities and the capacity to perform activities of daily living (ADLs) independently. Median survival time after the diagnosis is estimated to be around 6 years (4).

Mild Cognitive Impairment (MCI) is a state of cognitive decline that is not severe enough to alter daily functioning and that would represent an inflection point on the continuum between normal cognition and dementia (110). The development of this concept has been motivated by the possibility of altering the course of the disease by identifying individuals presenting early signs of cognitive decline and offering them effective interventions. Its prevalence is estimated to range between 15% and 20% in adults 60 and older. While not all individuals who have MCI develop AD, between 8% and 15% progress to dementia each year (110).

Women are more likely to develop AD than men, especially after the age of 80, which is thought to be linked with women’s longer life expectancy, but also with hormonal and genetic factors, and gendered occupational roles and opportunities (4, 111). Heritable factors account for 60 to 80% of the risks of developing AD. While the APOE ε4 allele is a well characterized genetic risk factor of AD, it does not account for all genetic risks of developing the disease. Other genes and proteins related to genetic risks of AD have been identified. Interestingly, they are involved in microglial response pathways, which supports a role for CNS immune response and neuroinflammation in the pathophysiology of AD (4).

Beta-amyloid accumulation in the brain is currently the most commonly accepted hypothesis for the onset of AD pathology, but findings that beta-amyloid is also found in the brain of healthy older adults and that the association between beta-amyloid load and dementia symptoms lessens with age support the contribution of other factors, such as tau accumulation, inflammation, as well as metabolic and cardiovascular factors (106, 112). Viral infections, notably with viruses from the *Herpesviridae* family, have been implicated in the pathogenesis of AD (113, 114). While viral infections are certainly not the only factor in the onset of the disease, they can interact with genetic, immune, and environmental factors and could contribute to neuroinflammation and beta-amyloid depositions, at least in some cases.

A systematic review of and meta-analysis of 11 original studies has found significantly reduced gut microbiota diversity in individuals with AD, but not MCI, when compared to controls (115). *Proteobacteria*, *Bifidobacterium* and *Phascolarctobacterium* were more abundant in individuals with cognitive decline (MCI and AD), whereas *Firmicutes*, *Clostridiaceae*, *Lachnospiraceae* and *Rikenellaceae* were less abundant when compared to controls. The increase of *Proteobacteria* and *Phascolarctobacterium* and the decrease of *Clostridiaceae* were associated with the progression of cognitive decline.

#### 4.1.1 Preclinical studies of AD

Preclinical studies of microbiome-based interventions in animal models of AD have been reviewed recently (116–118). The majority of these studies have been conducted in mice, including transgenic models of AD (APP/PSI, 3xTg-AD, SAMP8, 5XFAD, and APP knock-in mice), mice in which AD-like symptoms have been induced by the injection of a specific substance (beta amyloid peptides, D-galactose, or scopolamine), and aged mice. Several studies were conducted in rats injected with a substance that induced AD-like symptoms or aged rats, and only a few studies were conducted in *Drosophila melanogaster* or *C. elegans*. Siripaopradit et al. (116) conducted a systematic review and meta-analysis of 21 studies published between 2016 and 2022. Using slightly different criteria, Neta et al. (117) included 17 studies published between 2014 and 2020 in their systematic review. Lastly, Meng et al. (118) found 40 studies published between 2012 and 2020 that were eligible for qualitative review, with a subset of 22 studies that were considered eligible for inclusion in the meta-analysis.

The systematic review and meta-analysis of Siripaopradit et al. (116) included studies that tested the effects of one or more strains of *Lactobacillus* spp., *Bifidobacterium* spp., or both, and summarized results categorized into four groups of outcomes: AD pathology, cognitive function, neuroinflammation, and gut microbiota composition. Different arms of the same study testing the effects of a specific probiotic strain were included separately in meta-analyses. In terms of AD pathology, the meta-analysis revealed a significant reduction in beta-amyloid deposition, but not tau hyperphosphorylation following probiotic supplementation. For cognitive function, they found a significant advantage for animals receiving probiotics in all three cognitive functions assessed: short-term memory (Y-maze), long-term memory (passive avoidance test), and spatial recognition (Morris Water Maze). Several markers of neuroinflammation were significantly reduced by probiotics, including TNF-α, IL-1, IL-6, and Iba1. BDNF, a marker of synaptic plasticity, was significantly increased. However, there was no significant difference in Glial fibrillary protein (GFAP). Lastly, gut microbiota changes were reviewed qualitatively based on results focusing on diversity and relative abundance reported in 11 studies. Four out of six studies measuring diversity found that probiotics increased alpha and/or beta diversity, while the other two reported no changes. Results at the phylum level included increases in the relative abundance of *Verrucomicrobia*, *Actinobacteria* and *Firmicutes* and decreases in *Proteobacteria* and *Bacteroidetes*.

The conclusion of the systematic review of Neta et al. (117) were globally consistent with those of the systematic review and meta-analysis of Siripaopradit et al. (116). Neta et al. (117) applied no restriction in terms of the probiotics used and included three studies that tested the effects of an *S. thermophilus* strain. Out of eight studies measuring beta-amyloid accumulation, six found that probiotics were able to prevent or reduce it. Probiotics led to significant improvements in spatial recognition, learning and memory processes, and general cognition. Decreases in TNF-α and IL-1β were found in all studies measuring these parameters. Other markers of inflammation were measured in smaller numbers of studies but changes tended to be positive in animals receiving probiotics overall. In all four studies measuring BDNF levels, probiotics increased the expression of BDNF or reversed its suppression. Only a few studies measured markers of oxidative stress (malondialdehyde (MDA), superoxide dismutase (SOD) and catalase) and reported mixed results. Lastly, all five studies that measured microbiota composition concluded that it was restored after probiotic supplementation.

Meng et al. (118) reported conclusions that were consistent with those of the two aforementioned reviews. The meta-analysis showed a significative advantage for animals receiving probiotics compared to controls on three experimental paradigms of cognitive function (Morris Water Maze, Y-maze, passive avoidance test). The qualitative review of other experimental paradigms (e.g., Novel Object Recognition) also supported cognitive benefits conferred by probiotic supplements. The qualitative review of biomarkers showed several positive effects, including a reduction of beta-amyloid accumulation, a reduction in the levels of pro-inflammatory markers such as TNF-α, a downregulation of autophagy and apoptosis, a reduction in markers of oxidative stress, an increase in BDNF levels, and the regulation of cholinergic transmission.

Overall, probiotics exert several positive effects, notably a significant reduction of beta-amyloid accumulation, which was also reported in a recent study using *L. rhamnosus* HA-114 and *B. subtilis* Rosell^®^-179 in *C. elegans* (119). These results suggest that probiotics may not only have risk reduction benefits but could also beneficially influence the rate and progression of the disease after its onset.

#### 4.1.2 Clinical studies in people with AD

With respect to clinical studies of microbiome-based intervention, AD is the NDD that has been the most studied, and for the longest. Coherent with this, an umbrella meta-analysis of 13 meta-analyses of RCTs on the effects of probiotics on cognitive and metabolic function in AD has recently been published (120). Individual meta-analyses aggregated in this umbrella meta-analysis included between three and 12 original RCTs (counting the study of Akhgarjand et al. (121) including two arms testing different strains, *L. rhamnosus* HA-114 and *B. longum* Rosell^®^-175, as two different studies as was done in the meta-analysis of Mo et al. (122)) that focused on the effects of probiotics supplementation, with some including investigations of synbiotics. These meta-analyses, which were published over a period of 4 years (2020–2024) and used slightly different selection criteria and search strategies, collectively included 24 different original RCTs. These studies were conducted in people with MCI or AD and comprised a total of 3,910 participants. Follow-up duration ranged from two to 24 weeks, and most studies used a 12-week intervention.

All included meta-analyses aggregated cognitive function outcomes based on tools such as the Mini Mental State Examination (MMSE) and the Repeatable Battery for the Assessment of Neuropsychological Status (RBANS), and several also aggregated biomarkers-related outcomes. The umbrella meta-analysis showed a significant improvement in cognitive function following probiotic supplementation. However, the subgroup analysis revealed that the improvement was not significant in participants with MCI. In terms of markers of oxidative stress and inflammation, they found a significant reduction in MDA and high-sensitivity C-reactive protein (hs-CRP) levels, a significant increase in total antioxidant capacity (TAC), but no effects on glutathione and nitric oxide. In terms of glycemic index and lipid profile, the analysis revealed a significant reduction in homeostatic model assessment for insulin resistance (HOMA-IR) and triglyceride levels, but no significant difference in the quantitative insulin sensitivity check index (QUICKI), total cholesterol levels, and very-low-density lipoprotein (VLDL) levels.

One limitation of the umbrella meta-analysis of Xiao et al. (120) is that it summarized measures of global cognition only. However, a 2024 meta-analysis included in this study provided a more detailed review of cognitive function comparing different global cognition assessments and cognitive domains (122). Consistent with the results of Xiao et al. (120), Mo et al. (122) found significant improvements in global cognition following probiotics supplementation. However, in this study, changes were significant for both participants with MCI and AD. The subgroup analysis revealed that a significant difference was found both in studies using the MMSE and studies using other tools. Using information extracted from specific tools or subscales, they also analyzed changes in several domains of cognition, and found significant benefits of probiotics for recall/delayed memory, attention, and visuospatial/constructional abilities, but no differences in orientation, registration/immediate memory, and language. Lack of change can be explained by the relative preservation of these cognitive domains in MCI and early AD, and by heterogeneity present within and across included studies.

Microbiome-based interventions appear to support cognitive functioning in individuals with AD. Benefits observed in global cognitive functioning and some specific domains of cognition are supported by modulation of biomarkers with overall neuroprotective effects. Mixed evidence for cognitive support in MCI may be related to heterogeneity across studies, notably in terms of the criteria and assessment tools used to identify MCI (122). They may also be related to assessments’ limited sensitivity to small improvements and ceiling effects. It is also worth noting that in the case of MCI, stability in cognitive performance, and not necessarily improvement, could represent a positive outcome. However, determining the stability of cognitive performance requires long follow-up periods, ideally with multiple data collection points, which is often not feasible.

Reviews published so far have not compiled the effects of microbiome-based interventions in mental well-being and quality of life in MCI and AD, which is an important gap (but see original studies like Akhgarjand et al. (121) that reports positive effects on anxiety). Depression has recently been included in a list of 12 modifiable risk factors of dementia, but other disorders like anxiety have also been implicated in its onset (123). In addition, neuropsychiatric symptoms (e.g., depression, anxiety, sleep disorders) are highly prevalent in AD and are increasingly recognized as a core feature of the disease (124). They have been associated with poorer prognosis, acceleration of disease progression, and early institutionalization, in addition to interfering with treatments (124). Meanwhile, growing evidence shows the benefits of microbiome-based interventions to support mental health (12), which could be leveraged for the management of some behavioral and mood symptoms in AD. This appears as a valuable goal for future research, since depression was significantly associated with a reduction in self-evaluated health-related quality of life (HRQoL) in individuals with AD, and anxiety showed a trend for a similar reduction in HRQoL (125).

### 4.2 Parkinson’s disease

Parkinson’s disease (PD) is the second most common NDD after AD. It is associated with the loss of dopaminergic cells in the substantia nigra, which is thought to result from Lewy body inclusions formed by alpha-synuclein aggregation (64). PD is a movement disorder characterized by tremor, bradykinesia (slowness of movement), rigidity, and postural instability. Several other symptoms can be part of the profile (e.g., cognitive decline, mood disorders, behavioral changes), contributing to the complexity and heterogeneity of PD. In contrast with other NDDs, people can live with PD for decades.

Stages of disease progression linking cell death with the emergence of clinical symptoms in a predictable sequence and pattern have been described by Braak et al. (126). Interestingly, in the same paper, Braak proposed that this succession of changes would be triggered by “neuroinvasion by an unknown pathogen.” While the stages described by Braak have received support in the literature, this latter element of the model remains controversial. Alpha-synuclein aggregates are thought to form initially in enteric and olfactory nerves, and to be potentially exacerbated by local infections (64). They would then spread through the vagus or olfactory nerve and reach the brain where they would have cytotoxic effects. This would initially trigger a beneficial immune response but would eventually lead to neuroinflammation. Gut dysbiosis and resulting systemic inflammation would also contribute to the disease process.

The incidence and prevalence of PD has increased substantially across the world over the past 20 years for reasons that remain largely unknown (11). Early symptoms of the disease can precede the diagnosis by several years, and include constipation (the most common early symptom), REM sleep disorder, hyposmia (smell impairment), pain, and depression (11). Risk of developing the disease increases with age, but around 25% of individuals with PD are diagnosed before the age of 65. Monogenic forms of PD represent a small proportion of cases and are associated with disease onset before the age of 40. Among identified risk factors, exposure to pesticides and traumatic brain injury have received substantial support in the scientific literature (11). Women are less likely than men to get the disease and tend to develop it later, and therefore to live with disability for a shorter period. They are also at a lower risk of developing PD-related cognitive decline. However, women appear to be at greater risk of developing medication-related complications, such as dyskinesia and response fluctuations, as well as experiencing urinary symptoms and depression.

As mentioned above, the potential role in the disease of bacteria involved in sulfur metabolism, notably *Desulfovibrio* spp., has recently been identified in preclinical studies (71) and increased abundance of these taxa had been described in studies of individuals with PD (68, 69, 72). Furthermore, multiple changes in the composition of the gut microbiota of individuals with PD have been identified (54). A recent study described three clusters that would summarize changes in the relative abundance of gut bacteria in PD: an increase in opportunistic pathogens (*Porphyromonas*, *Prevotella*, and *Corynebacterium*), a decrease in SCFA producers (e.g., *Faecalibacterium*, *Roseburia*), and an increase in bacteria commonly used for their probiotic properties (*Lactobacilli* and *Bifidobacteria*) (127). The authors measured several cofounding factors, including factors related to the diet (e.g., fruits and vegetable intake). The increase in *Lactobacilli* and *Bifidobacteria* may reflect a response to the rise in opportunistic pathogens, the decrease in SCFA producers, or be linked to dopamine replacement therapy (levodopa) metabolism.

#### 4.2.1 Preclinical studies of PD

Animal models of PD include rodents, non-human primates, and non-mammalian species (e.g., drosophila, *C. elegans*, zebra fish) in which PD-like symptoms are induced by the administration of various neurotoxins (e.g., MPTP, 6-OHDA, rotenone, paraquat), or by genetic mutations (128). As PD is a complex and heterogeneous disease, it is not surprising that none of these animal models are able to reproduce its full symptomatology (54, 128). Certain species and symptom induction methods are selected to investigate the effects of microbiome-based interventions on specific mechanistic pathways or symptoms of the disease. Nevertheless, comprehensive reviews of preclinical studies of microbiome-based interventions in PD have been published (129–131).

A 2024 comprehensive review (131) included a total of 25 preclinical studies on probiotics, 7 studies on engineered probiotics, 6 studies on probiotics administered with another substance (e.g., vitamin B6, prebiotics), and 7 studies focusing on prebiotics. The majority of studies reviewed have been conducted in mice in which PD-like symptoms have been induced using neurotoxins and have investigated the effects of *Lactobacilli*, especially *L. plantarum*, *L. rhamnosus*, and *L. acidophilus* strains. Overall, studies of probiotics, synbiotics, and prebiotics in PD models have shown a vast range of neuroprotective effects and health benefits, and are aligned with clinical studies conducted to date. Observed neuroprotective effects included preservation of dopaminergic cells, reduction of α-synuclein aggregation, reduction of oxidative stress and inflammation, and restoration of neurotrophic pathways. Some studies also observed effects on mitochondrial autophagy (132). In terms of PD-like symptoms, supplements were shown to exert positive effects on both motor and non-motor symptoms, such as cognitive function. For example, *L. rhamnosus* HA-114 rescued hippocampus-dependent cognition in Sprague-Dawley male rats (133). Overall, results reviewed by Hor et al. show coherence and consistency across animal models, which lends additional confidence in findings (117).

#### 4.2.2 Clinical studies in people with PD

About a dozen clinical studies on microbiome-based supplementation have been published to date and have been included in a recent comprehensive review (131) and a systematic review with meta-analysis (134). The majority of studies have investigated the effects of supplements comprising *Lactobacilli* and *Bifidobacteria*, and only a handful of studies have investigated prebiotics and synbiotics (131). As highlighted in these papers, most of the studies published to date have focused on the potential of microbiome-based interventions to support gastrointestinal comfort, especially to alleviate constipation. Some studies have documented effects on motor symptoms, functional ability, and support to standard dopamine replacement therapy (e.g., reduction of OFF-state), and have explored the potential benefits of these interventions for mental health (depression, anxiety) and cognitive function.

The systematic review and meta-analysis of Jin et al. included 11 RCTs on the effects of probiotics collectively including a total of 756 individuals with PD (134). There was substantial heterogeneity among studies measuring common outcomes, which can be attributed at least in part to the heterogeneity inherent to PD. The meta-analysis showed significant benefits for participants receiving probiotics compared to placebo for the frequency of complete bowel movements (CBM) and on the Patient Assessment of Constipation Quality of Life Questionnaire (PAC-QOL). However, there was no significant improvement for stool consistency measured with the Bristol Stool Scale (BSS), defecation effort, or severity of motor symptoms (MDS-UPDRS III), despite positive effects reported in individual studies.

Only a few studies of microbiome-based interventions have reported mental health and cognitive function outcomes. Sun et al. (135) recruited 82 participants with PD who were randomly assigned to receive a probiotic (*Bifidobacterium animalis* subsp. *lactis*) or placebo in addition to their regular medication for 3 months. The intervention modulated the composition of the gut microbiota and increased the abundance of taxa producing key metabolites, including GABA and SCFAs. One month into the intervention, probiotic participants had significantly improved sleep quality (Parkinson’s Disease Sleep Scale, PDSS) compared to placebo participants. The between-group difference was not significant after 3 months, although the within group comparison was significant in the probiotic group only. At the end of the intervention, participants receiving the probiotic had significant improvement compared to the placebo on the Hamilton Anxiety Scale (HAMA). Both groups had significant progress from baseline to the end of the intervention in measures of depression (Hamilton Depression Scale-17, HAMD-17) and cognitive function (Mini Mental State Examination, MMSE). Other significant advantages for the probiotic over the placebo included enhanced stool consistency (BSS) and improved constipation-related quality of life (PAC-QOL).

Yang et al. (136) studied the effects of supplementation with *Lacticaseibacillus paracasei* in fermented milk. In this study, 128 individuals with PD were randomized to receive probiotic fermented milk or placebo for 12 weeks. Although the authors found no major changes in gut microbiota composition or fecal metabolites at the end of the intervention, they observed significant improvements in the probiotics group compared to the placebo on two measures of non-motor symptoms of PD, the Movement Disorder Society Unified Parkinson’s Disease Rating Scale (MD UPDRS I) and the Non-Motor Symptoms Scale (NMSS). These scales cover multiple dimensions of non-motor symptoms, including sleep, fatigue, depression, anxiety, cognition, but also gastrointestinal function, constipation, pain, and weight changes. Furthermore, the authors found significant improvements in the probiotics group compared to the placebo on the HAMD-17 and the HAMA, but not on measures of global cognition (MMSE; Montreal Cognitive Assessment, MoCA). They further reported significant improvements compared to the placebo in a measure of functional abilities in activities of daily living (Parkinson’s Disease Questionnaire, PDQ-39) and several measures of gastrointestinal comfort, including constipation severity (Wexner score), stool consistency (BSS), number of bowel movements, and constipation-related quality of life (PAC-QOL).

Becker et al. (137) studied the effects of an eight-week prebiotic intervention using resistant starch to modulate fecal levels of SCFAs. They enrolled 32 people with PD and 30 controls who received resistant starch supplementation, and 25 individuals with PD who only received dietary instructions. In the PD group receiving resistant starch, but not the PD group receiving dietary instructions, significant improvements were observed from baseline to the end of the intervention in non-motor symptoms (NMSS) and depression scores (Beck Depression Inventory). However, no changes were noted in bowel habits measured with the Constipation Scoring System (CSS). In terms of biomarkers, this group also exhibited a significant reduction in intestinal inflammation (decreased calprotectin) and increased fecal levels of butyrate.

Microbiome-based interventions significantly improve gastrointestinal comfort and alleviate constipation in PD. Considering this, it is possible that improvements on composite scales of non-motor symptoms may be driven by improvements in the management of gastrointestinal symptoms and constipation. However, improvements on measures of sleep, depression, and anxiety reported in recent studies suggest that benefits extend beyond gastrointestinal symptoms. The investigation of those important aspects of the disease contrasts with studies of AD where they have largely been overlooked. Results on the improvement of cognitive function and motor symptoms are more mixed. In terms of cognition, only a few studies using global cognition evaluation tools have investigated this aspect to date. The use of more specific evaluations, especially tools that target cognitive domains known to be affected more prominently and earlier in PD (e.g., executive functions) could help identify and characterize the effects of probiotics on cognition in PD. The evaluation of motor symptoms with the MDS-UPDRS III was reported in only three studies included in the meta-analysis of Jin et al. (134). While two out of three showed modest but positive results for the probiotics, the overall effect was not significant in the meta-analysis. Further studies with larger samples and well-balanced groups in terms of age, disease duration, and proportion of men and women would be needed to better appraise this effect.

### 4.3 Multiple sclerosis

Multiple Sclerosis (MS) is an autoimmune and inflammatory disorder that results in demyelination of nerve cells in the brain, spinal cord, and optic nerve (9, 138). Common symptoms include fatigue, muscle weakness, numbness, loss of balance, and vision impairments. Other symptoms include behavior changes, psychological disorders (depression, anxiety), and cognitive decline. Bowel dysfunction is also common and consists mostly of constipation but can also include diarrhea and incontinence (9, 138).

MS is typically diagnosed between the ages of 20 and 30 but it can also be diagnosed in children and in adults over the age of 30 (9, 73, 138). The etiology of MS is related to immune, hormonal, genetic, and environmental factors. Fungal, bacterial, or viral infections have also been implicated in the disease, notably Epstein-Barr virus infections (9, 73, 138, 139). Immune triggers of the disease were originally attributed to a T-mediated response, but lymphocytes B and microglia have also been involved. The prevalence of MS is higher in women than men, a characteristic shared by multiple autoimmune diseases (53, 140). This is thought to be related to hormonal, genetic, and environmental or lifestyle factors. MS epidemiology is also characterized by a latitude gradient, whereby people living away from the equator are more susceptible to getting the disease. This has been related to sunlight exposure and vitamin D metabolism (9, 138).

The course of MS varies across individuals (9). Relapsing-remitting MS (RRMS) is characterized by the alternance between episodes of neurological symptoms and periods of complete or nearly complete remission. Over time, most people with RRMS will transition to secondary progressive MS (SPMS) which is characterized by progressive worsening of symptoms with few clear episodes of relapse or remission, but some possible plateaus. About 15% of individuals with MS have primary progressive MS (PPMS) and experience progressive worsening of symptoms from disease onset.

Several taxa, including *Pseudomonas*, *Mycoplana*, *Haemophilus*, *Blautia*, *Dorea*, *Faecalibacterium*, *Methanobrevibacter*, *Akkermansia*, and *Desulfovibrionaceae* have been found to be more abundant in MS patients than controls (73). While an increase in *Akkermansia* is related with the disease, it has been proposed to represent a compensatory mechanism for the reduced abundance of beneficial species.

#### 4.3.1 Preclinical studies of MS

Experimental autoimmune encephalomyelitis (EAE) is the most commonly used preclinical model of MS. The possibility of modulating the EAE phenotype has been investigated in studies of microbiome-based interventions. Most studies published to date explored the effects of probiotics, with a few studies exploring the effects of prebiotics. A recent systematic review and meta-analysis (141) included 17 studies in EAE rodents (16 in mice, one in rats) testing the effects of probiotics on several biomarkers. The majority of studies tested the effects of a single strain of *Lactobacillus*, *Bifidobacterium*, *Enterococcus*, or *Streptococcus* spp. The meta-analysis showed that probiotics decreased levels of IFN-γ, IL-17, and TNF-α, increased levels of IL-10, modulated numbers of T-reg CD4/CD25 cells, and improved the animal equivalent of clinical scores.

A 2021 systematic review and meta-analysis of 15 studies in EAE rodents (eight in mice, seven in rats) that focused on disease trajectory outcomes found that probiotics reduced the incidence and delayed the onset of symptoms after EAE induction, supported normal weight gain, and attenuated the animal equivalent of clinical symptoms (142). A reduction in the duration of the disease was only found in two studies using supplementation with *Enterococcus faecium*. The meta-analysis revealed a significant reduction in mortality in female animals only (142).

In terms of gastro-intestinal comfort management, Legan et al. (143) studied the effects of *B. subtilis* Rosell^®^-179 on gastrointestinal dysmotility and constipation in EAE mice. After a week of probiotic supplementation, EAE mice had significantly faster colonic motility times than vehicle-treated animals.

Prebiotics have been investigated in three studies in EAE mice. Inulin modulated the composition of the gut microbiota and increased the concentration of butyric acid (144). It ameliorated the severity of symptoms, reduced the concentration of pro-inflammatory cytokines (IL-17, IL-6, TNF-α), and reduced demyelination in the CNS, in addition to decreasing the proportion and numbers of pathogenic Th17 cells in the brain and spleen. Ellagic acid, a polyphenol naturally rich in the Mediterranean diet, modulated the composition of the gut microbiota and increased the relative abundance of SCFA producers, halting the progression of symptoms (145). Pomegranate peel extract modulated the composition of the gut microbiota, relieved symptoms, and attenuated CNS inflammation and myelin loss (146).

#### 4.3.2 Clinical studies in people with MS

A few clinical studies and secondary analysis studies investigating probiotics, prebiotics, and/or synbiotics have been published to date (147–156). Kouchaki et al. (147) conducted a randomized, double-blind, placebo-controlled trial in 60 participants with MS randomly assigned to receive a probiotic supplement (*L. acidophilus*, *L. casei*, *B. bifidum*, and *L. fermentum*) or placebo for 12 weeks. After 12 weeks, participants receiving the probiotic had better scores on the Expanded Disability Status Scale (EDSS) and measures of mental health symptoms compared to participants taking the placebo. The probiotic modulated inflammatory factors and oxidative stress biomarkers, and exerted significant positive effects on insulin and cholesterol metabolism. The same research group (150) tested the effects of a different probiotic formulation (*B. infantis*, *B. lactis*, *L. reuteri*, *L. casei*, *L. plantarum* and *L. fermentum*) in 48 participants with MS receiving the probiotic or placebo for 16 weeks. Similar to their previous study, they observed better scores on the EDSS and mental health measures in participants taking the probiotic. The probiotic was also associated with improvements in inflammation and oxidative stress markers, including significant reductions in IL-6 and hs-CRP, and significant increases in IL-10 and nitric oxide levels, along with a trend toward improved markers of insulin metabolism.

Tankou et al. (148, 149) provided individuals with MS (*n* = 9) and controls (*n* = 13) with a supplement comprising *L. paracasei*, *L. plantarum*, *L. acidophilus*, *L. delbrueckii* subsp. *bulgaricus, B. longum*, *B. infantis*, *B. breve*, and *S. thermophilus* twice daily for 2 months. In MS participants, the probiotic increased the abundance of *Lactobacillus* species and decreased the abundance of *Akkermansia* and *Blautia* compared to baseline. Predictive metagenomics analysis revealed a modulation of several gut-microbiota-related metabolic pathways known to be altered in MS (e.g., methane metabolism). The probiotic also induced anti-inflammatory peripheral immune response on monocytes and dendritic cells. In controls, probiotic supplementation was associated with decreased expression of MS risk allele HLA-DQA1.

Rahimlou et al. (151, 152) recruited 70 participants with MS to receive a probiotic supplement comprising 14 strains or placebo twice daily for 6 months. After 12 weeks, they observed a significant reduction in serum concentration of inflammatory biomarkers including CRP, TNF-α and IFN-γ, as well as an increase in level of anti-inflammatory factors, including TGF-β1 and FOXP3 (152). After 6 months, the probiotic group had a significant increase in BDNF levels and a significant decrease in IL-6 levels compared to the placebo (151). The probiotic group also had significantly better scores than the placebo group in measures of general health, depression, fatigue, and pain.

The study of Asghari et al. (153) involved 40 participants who received a supplement of *Saccharomyces boulardii* or placebo for 4 months. Compared to the placebo, the probiotic group showed significant gains on the somatic and social dysfunction subscale of the General Health Questionnaire. The probiotic group also had a significant improvement in quality of life in some subscales of 36-Item Short Form Survey, in pain intensity, and in fatigue severity compared to the placebo. Furthermore, the probiotic significantly decreased levels of hs-CRP, and increased serum antioxidant capacity.

Moravejolahkami et al. (154, 155) randomly assigned 70 participants with progressive forms of MS to receive a synbiotic supplement (*L. casei*, *L. acidophilus*, *L. plantarum*, *L. bulgaricus*, *B. breve*, *B. infantis*, *B. longum*, *S. thermophilus*, and fructooligosaccharide) and an anti-inflammatory-antioxidant-rich diet, or placebo with dietary recommendations for 4 months. Compared to the placebo, the synbiotic and dietary intervention led to significant improvements in measures of fatigue, pain, bladder and bowel control, and sexual function (154). The intervention was also effective at reducing levels of fecal calprotectin, a marker of intestinal inflammation (155).

Using a cross-over design, Straus Farber et al. (156) tested the effects of a six-week probiotic (*Lactobacillus, Bifidobacterium and Streptococcus* spp.) and prebiotic (oligofructose enriched inulin) supplementation in 22 individuals with MS. An additional group of 15 participants received prebiotics supplements only. When comparing baseline scores to those obtained after supplementation, the only significant change observed was an improvement in bowel control, as measured by the bowel control scale (BWCS) following probiotic supplementation. This study’s protocol had to be modified due to interruptions caused by the COVID-19 pandemic which may have affected the ability to capture significant changes.

Overall, studies show that biotics interventions contribute to the management of several MS symptoms including bladder and bowel control, depression, fatigue, and pain. However, variability in outcomes and measures used across studies limits comparability. Eight weeks of supplementation appears sufficient to induce observable changes in biomarkers. However, shorter intervention durations may be insufficient to induce measurable changes on several clinical measures.

### 4.4 Amyotrophic lateral sclerosis

Amyotrophic lateral sclerosis (ALS) is a rare neurodegenerative disorder that results from the progressive loss of upper and lower motor neurons that control voluntary movement (10). ALS presents as different phenotypes, with the spinal and bulbar phenotypes being the most common. The spinal phenotype, or classical ALS phenotype, is characterized by symptoms commencing in the limbs. Bulbar onset ALS is characterized by early speech (dysarthria) and swallowing (dysphagia) symptoms. Symptoms typically progress rapidly, and median survival is estimated to range between 2 and 4 years.

The prevalence of ALS varies across age, sex, and genetic (racial, ethnic) groups (10). While the majority of cases have no genetic basis, about 10% to 15% of ALS cases are inherited. ALS was traditionally considered as a motor dysfunction, but the presence of cognitive, psychological, emotional, and behavioral changes early in the course of the disease is increasingly recognized. As such, it is currently conceptualized as being on a continuum with frontotemporal dementia (FTD), a NDD primarily characterized by cognitive and behavioral symptoms (157). The presence of TDP-43 proteinopathy is thought to subtend this association (10, 157).

Pathophysiological processes of ALS have not been fully elucidated, but they include aggregation of RNA/DNA binding proteins (TDP-43 and FUS) into inclusions, which impairs RNA processing; impaired clearance of damaged proteins, leading to dysregulation of proteostasis and autophagy; cytoskeletal and tubulin defects that block the movement of cellular components along axons (axonal trafficking); and mitochondrial dysfunction leading to oxidative stress (10). ALS proteinopathy could also be propagated in the body in a prion-like fashion. All these phenomena would contribute to, and could be exacerbated by, local and systemic inflammation. Accumulation of risk factors through aging and cumulative lifetime exposure to a variety of environmental factors (the ALS exposome (158)) would trigger the physiological changes that ultimately lead to ALS symptoms. Viral and fungal infections have been linked with ALS, but exposure to Cyanobacteria in low-quality bodies of water has received more attention and support in the literature (158, 159).

Early studies of the gut microbiota composition of ALS patients have yielded mixed results, with some finding marked alterations compared to healthy individuals (160, 161), changes when comparing patients with more severe symptoms to controls (159), or no substantial changes (162). More recent studies that divided patients by clinical phenotypes (spinal and bulbar ALS) have identified different patterns of gut microbiome alterations (163, 164). Fontdevila et al. (163) found no significant differences in alpha diversity in ALS patients compared to controls overall, but observed a higher abundance of *Enterobacter*, *Clostridium*, *Veillonella*, *Dialister*, *Turicibacter*, and *Acidaminococcus* species, and lower abundance of *Prevotella*, *Lactobacillus*, and *Butyricimonas*. They also found increased *Fusobacteria* and *Tenericutes* in spinal ALS compared to bulbar ALS, and different patterns of correlation between phyla when comparing ALS patients and controls, and when comparing ALS phenotypes. Kim et al. (164) found significant gut dysbiosis in spinal ALS and significant oral dysbiosis in bulbar ALS. The association with dysbiosis of one specific body ecosystem and ALS phenotype was also correlated with the severity of symptoms.

#### 4.4.1 Preclinical studies of ALS

A small number of studies using various *in vitro* and *in vivo* preclinical models of ALS have been published to date. It is worth noting that those results apply to spinal ALS only, since no preclinical models of bulbar ALS (e.g., dysphagia) have been developed (164). This represents a potential caveat for the translation of preclinical into clinical research, which is further emphasized by the well-documented differences in responses to medication across ALS phenotypes (164).

To mitigate elevated levels of interferon-gamma (IFN-γ) observed in ALS, Chen et al. (165) tested the capacity of three probiotic formulations to modulate the secretion of IFN-γ by natural killer (NK) cells, CD8+ T cells, and peripheral blood mononuclear cells *in vitro*. They found differential profiles across formulations, with the formulation designed to target immune modulation yielding the greatest reduction in IFN-γ secretion in cells challenged with IL-2.

Using a *C. elegans* model of ALS, Labarre et al. (166) found that *Lacticaseibacillus rhamnosus* HA-114 exerted neuroprotective effects demonstrated by its ability to rescue motor defects and paralysis in transgenic nematodes. Beneficial effects of *L. rhamnosus* HA-114 were subtended by its ability to restore lipid metabolism homeostasis and energy balance through mitochondrial β-oxidation.

Song et al. (167) tested the effects of galactooligosaccharides (GOS), a prebiotic that could promote vitamin absorption to support homocysteine metabolism in ALS. GOS and GOS-enriched yoghurt significantly delayed disease onset and rate of disease progression in SOD1G93A mice. The intervention attenuated motor neuron loss, improved mitochondrial function in skeletal muscles and reduced atrophy, in addition to regulating several inflammatory- and apoptosis-related factors and suppressing the activation of astrocytes and microglia. Another study in SOD1G93A mice tested the effects of a probiotic supplement comprising *Lactobacillus acidophilus*, *Bifidobacterium longum*, and *Enterococcus faecalis* (168). The probiotic supplement modulated the intestinal microbiota, increased SCFA levels and enhanced autophagy flux. It attenuated the proinflammatory response, reduced aberrant superoxide dismutase 1 (SOD1) aggregation, and protected enteric and spinal neuronal cells. A follow-up study by the same group further elucidated the contribution of SCFA to the mitigation of ALS symptoms and showed that systemic administration of butyrate and propionate produced similar benefits as probiotic supplementation (169).

Blacher et al. (161) identified 11 taxa associated with ALS severity in *Sod*1-Tg mice. They supplemented antibiotic-treated *Sod*1-Tg mice with each of these taxa and found that *Akkermansia muciniphila* ameliorated symptoms, while *Ruminococcus torques* and *Parabacteroides distasonis* worsened symptoms. The beneficial effects of *A. muciniphila* were associated with the accumulation of nicotinamide in the CNS, which was further supported by an improvement in motor symptoms and gene expression patterns in the spinal cord of *Sod*1-Tg mice following systemic supplementation with nicotinamide.

In TDP43 mice, daily supplementation with a probiotic formulation comprising *Lactobacillus acidophilus*, *Lactobacillus plantarum*, *Lactobacillus paracasei*, *Lactobacillus helveticus*, *Bifidobacterium breve*, *Bifidobacterium longum*, *Bifidobacterium infantis*, and *Streptococcus thermophilus* resulted in improved motor function, enhanced enteric nervous system function, increased intestinal motility and decreased permeability (170). Probiotics modulated the expression of several genes, reduced cytokine secretion, and reduced aberrant protein aggregation. Similar results were observed with butyrate supplementation.

#### 4.4.2 Clinical studies in people with ALS

At the time of writing, only one clinical study has investigated the effects of probiotics in ALS. Di Gioia et al. (159) provided a daily placebo or probiotic supplement comprising five lactic acid bacteria (*Streptococcus thermophilus*, *Lactobacillus fermentum*, *Lactobacillus delbrueckii* subsp. *delbrueckii, Lactobacillus plantarum*, and *Lactobacillus salivarius*) to 50 participants with ALS who were followed longitudinally over a period of 6 months. Although the probiotic supplement modulated the composition of gut microbiota, it was not effective at restoring control-like composition and it had no influence on the progression of the disease as measured by the ALS Functional Rating Scale–Revised (ALSFRS-R). The study was limited by high attrition, high interindividual variability and limited collection of lifestyle measures, and the impossibility of performing subgroup analyses of ALS phenotypes.

The number of both preclinical and clinical studies focusing on elucidating the effects of microbiome-based interventions in ALS is very small, which may be explained at least in part by the lower prevalence of this condition compared to AD and PD. The existence of distinct phenotypes, and the fact that no animal model of the bulbar phenotype exists, combined with well-documented differences in response to medications across phenotypes in humans, further complexifies the investigation of the potential benefits of microbiome-based interventions in this condition. Nevertheless, recent developments in the understanding of ALS pathophysiology and promising preclinical results motivate the pursuit of investigations on microbiome-based interventions for the improvement of ALS symptoms.

### 4.5 Huntington’s disease

Huntington’s disease (HD) is a genetically transmitted NDD characterized by motor, cognitive, and psychiatric symptoms (171). It is caused by a mutation of the huntingtin (HTT) gene on chromosome 4 that results in the production of a mutant huntingtin protein (mHTT) that is expressed in the whole body. In the brain, mHTT causes neuronal dysfunction and death through various mechanisms including formation of abnormal protein aggregates and disruption of proteostasis, and resulting impairments in synaptic, mitochondrial, and axonal transport functions. Since the disease process is centered around the impacts of the mHTT protein, some have suggested that HD would be one of the NDDs most amenable to the development of a genetic treatment (171). This may explain why less research on the gut microbiome has been conducted in HD than in other NDDs, although recent studies point to the role of gut dysbiosis in disease progression and support the exploration of microbiome-based interventions (172).

Kong et al. (173) studied the gut microbiome in the R6/1 transgenic mouse model of HD compared to wildtype mice. They found an increase in *Bacteroidetes* and a proportional decrease in *Firmicutes* in HD animals. They also found an increase in microbial diversity in male HD mice, but not female HD mice. Gut dysbiosis was associated with lower body weight gain despite higher food intake, motor deficits, and higher fecal water content. Labarre et al. (166), who found a neuroprotective effect of *Lacticaseibacillus rhamnosus* HA-114 in a *C. elegans* model of ALS, further extended their findings to *C. elegans* models of HD. Indeed, they found that *L. rhamnosus* HA-114 rescued paralysis phenotypes in transgenic worms expressing pan-neuronal polyglutamine repeats. Modulation of gene expression of acdh-1 and kat-1 involved in fatty acid metabolism and β-oxidation was identified as the main mechanism subtending neuroprotective effects across models.

Rosas et al. (174) analyzed the plasma metabolomic profiles of 52 presymptomatic HD participants (premanifest HD), 102 early symptomatic HD participants, and 140 controls. They identified changes in tyrosine, tryptophan, and purine pathways in HD, many of which are related to cellular metabolism and oxidative stress (174). Furthermore, many metabolites distinguishing metabolomes between HD participants and controls were strongly related to the gut microbiome, suggesting disease-related changes.

Wasser et al. (175) studied the gut microbiota composition of HD gene expansion carriers (HDGECs) and age- and sex-matched controls. About half of the HDGEC participants had started experiencing symptoms recently (early manifest HD) while the other half had no symptoms at the time of the study (premanifest HD). Compared to controls, HDGEC participants had reduced alpha diversity (richness and evenness) and beta diversity. Interestingly, the authors found differences in the relative abundance of several taxa in male participants only, which is coherent with sex differences in the mouse model of HD reported by Kong et al. (173). Furthermore, Wasser et al. found no difference between early manifest and manifest HD participants except for a difference in the abundance of bacteria from the *Ruminococcaceae* family in male participants only. In terms of association with disease symptoms, they found a correlation between lower abundance of *Eubacterium hallii* and increased severity of motor signs in HDGEC, and higher probability of developing symptoms in the next 5 years in premanifest participants. The same group conducted a placebo-controlled randomized trial to test the effects of a 6-week probiotic intervention in HDGECs participants and controls (176). The probiotic supplement, comprising *Lactobacillus rhamnosus* LGG, *Saccharomyces cerevisiae* var. *boulardii*, and *Bifidobacterium animalis* subsp. *lactis*, had no impact on gut dysbiosis, cognition, mood, and gastrointestinal symptoms.

The scarcity of studies on gut microbiota changes and microbiome-based interventions in HD may be related to the existence of other research priorities, such as the development of genetic treatments, and the lower prevalence of HD compared to other conditions. The few studies that have focused on HD so far reveal the existence of gut microbiome changes and dysbiosis compared to healthy individuals. Those findings support the exploration of microbiome-based interventions as a potential solution to modulate pathways involved in the disease process, especially to alleviate symptoms and delay disease progression. In the absence of genetic treatments, exploring the contribution of microbiome-based interventions for the regulation of cellular metabolism and oxidative stress is a relevant objective.

## 5 Knowledge gaps and open questions

Currently, the factors that trigger neurodegeneration or any preceding changes that are directly linked with the onset of NDDs remain unknown (34, 106). However, key commonalities in physiological changes related to disease onset are starting to emerge. For the most part, hypotheses related to these factors are not entirely new (32, 113, 114, 126), but recent developments across several fields generate renewed interest (139). A better understanding of opportunistic pathogens (pathobionts), host factors that lead to colonization and pathogenic effects, and effects of infections throughout life and during aging (47) may help elucidate the long-observed association between pro-inflammatory immune response and NDDs.

Investigating the effects of microbiome-based interventions on the composition of the gut microbiota and on the metabolic activity of gut bacteria by relying on the analysis of feces samples presents several limitations (109). Gut bacteria metabolic activity, such as fermentation of dietary fibers and synthesis of SCFA, happens in the small intestine. Therefore, interpretation of results on the function of SCFAs and other bacterial metabolites based on the content of feces is likely biased or incomplete (41). To address this limitation and enable more accurate assessments, novel non-invasive technologies have been developed to collect samples directly from the small intestine (177). Omics and multiomics studies are also invaluable to elucidate the functions of the gut microbiome, and their influence on health and disease (102, 178, 179).

Knowledge gained from animal model studies brings an important contribution to the understanding of disease processes and modulations accomplished by microbiome-based interventions. However, preclinical models always constitute approximations and simplifications, and therefore, results should be interpreted with caution (4, 180). Furthermore, the translation of findings from animal models to humans is limited when it comes to understanding high-level cognition and behavior, such as executive functions, memory, and language, but also emotion regulation and mental health. No matter the complexity of behavior observed, rodent models present limitations for the study of gut microbiota due to the differences that exist between rodents and humans in anatomy, genetics, physiology, and influence of health history and lifestyle factors (181).

The prevalence of several NDDs varies according to sex, sometimes in interaction with other factors such as age, race, and ethnicity. Sex differences have gained more attention in studies of the pathophysiology of NDDs, especially with respect to understanding the role of hormones and their influence over the development of the nervous system and the MGBA (172, 182, 183). However, women remain underrepresented in clinical trials, and most preclinical studies continue to include only male animals (172, 184), leading to a lack of knowledge regarding the specificities of disease processes in women (172). Furthermore, in clinical studies, sex differences cannot be completely teased apart from gender differences, which are often closely linked to lifestyle and occupational risk factors that are also associated with the onset of NDDs. Even with large scale populational and longitudinal studies, gaining a better understanding of these factors and their interactions remains a challenge (111, 158).

This review has highlighted the pivotal role of the gut microbiome and of gut dysbiosis in the onset and progression of NDDs. However, emerging evidence suggests that imbalances in other body ecosystems may also contribute to the complex pathophysiology of these diseases. Notably, oral dysbiosis and proliferation of pathobionts has been found in AD and PD and points toward a role of pathogens such as *Porphyromonas gingivalis* and *Treponema denticola* in the disease process (48, 114, 185, 186). Interestingly, periodontitis, an inflammatory oral condition affecting the gingiva, has been identified as a non-traditional modifiable risk factor of cardiovascular diseases (187), which are also linked with NDDs. Relatedly, edentulism (tooth loss) has been associated with cognitive decline (188). Furthermore, a study found an association between gut dysbiosis and the spinal phenotype of ALS, and oral dysbiosis and the bulbar phenotype (164). In addition to the oral microbiome, researchers have also highlighted the potential contribution of microorganisms from other body cavities, such as the nasal (49, 66, 73) and pulmonary cavity (73) to NDD process. With respect to the nasal cavity, the olfactory system has been identified as a transmission route to the CNS for several viruses and as the initial site of alpha-synuclein pathology in some cases of PD (126, 189, 190). Another potential yet rarely discussed contribution is that of the vaginal microbiota (191), whose composition fluctuates along with changes in hormonal levels periodically (during menstruations) and in key stages of life (e.g., puberty, pregnancy, menopause) (192). Considering the existence of sex differences in the prevalence of NDDs, the role of hormonal factors in disease process, and gut bacteria functions in estrogen deconjugation (collectively known as the estrobolome) (191), these aspects would deserve more attention (172).

Research published so far has focused on the role of bacteria, but it is important to note that the gut is an ecosystem also composed of fungi, viruses, and other microorganisms (193–195). Recent studies suggest that gut fungi play important roles in the maintenance of gut homeostasis and in immunity but could also constitute a reservoir of opportunistic pathogens involved in several diseases (194). Importantly, gut fungi would also be implicated in the MGBA through their roles in the immune pathway, and in the production of various mediator compounds (195). Studies have started describing the implication of gut fungi in neurological disorders, including AD, PD, and MS (193). Furthermore, in addition to bacterial infections, fungal and viral infections have been identified as potential contributors to the disease process in some NDDs (139, 158).

An important open question is that of the interactions between the gut microbiome and medication (73, 127, 196, 197). Indeed, beyond the impact of the microbiome in physiological changes and onset of diseases, there would be several interactions that influence drug uptake and metabolism, with impacts on drug response and dosage, and potentially, disease progression. Studies in patients at different stages of the disease, especially studies included newly diagnosed individuals, would help clarify the interactions between the gut microbiome and medication.

## 6 Future research priorities

The interaction between MGBA and NDD pathology is complex, multifaceted, and subject to multiple sources of variation. To better understand the MGBA’s involvement in disease development and progression, future research should focus on integrative approaches that combine high-resolution molecular profiling with clinically meaningful outcomes. The use of multi-omics technologies (e.g., metagenomics, metabolomics, transcriptomics, and proteomics) could help clarify mechanistic links between the microbiome and host neurological health (102, 178, 179). For example, integrating multi-omics in the analysis of CSF or plasma samples could help identify microbial metabolites that cross the blood-brain barrier and modulate neuroinflammatory processes, and that correlate with clinical phenotypes. Open-access platforms such as MGnify and the Human Microbiome Project now provide reference frameworks for such integrative datasets. This enables harmonization of methodologies across research sites and supports reproducibility (198).

The use of advanced *in vitro* models or microphysiological systems that simulate the gut-brain axis more accurately than traditional animal models represents another promising avenue (199). For example, organ-on-a-chip (OoC) technology, provides a more controllable environment than traditional animal models and enables studying soluble microbial factors (e.g., neuroactive metabolites, lipopolysaccharides, and SCFA) and their direct and indirect effects on the intestinal barrier, immune system, BBB, and brain tissue (200). Interestingly, these micro-organs can also be microfluidically linked to form multi-organ platforms, offering a more integrative model. In addition to biochemical signals, important biomechanical parameters can also be studied including peristalsis-like motion and interstitial flow. In the context of MGBA and NDDs, brain and BBB-on-a-chip models have successfully represented complex features of the MGBA, including endothelial tight junction integrity and neuron-glia interactions, and are now being coupled with gut and immune compartments to study signals relevant to neurodegeneration. The MINERVA project exemplifies this interdisciplinary vision by aiming to integrate five functionally distinct compartments: microbiota, gut epithelium, immune cells, BBB, and brain, into a connected platform, thereby enabling the stepwise exposure of neuronal tissue to microbe-derived products under near-physiological conditions (201). These new technologies present challenges, including long-term cell viability, standardization across labs, and incorporation of patient-derived microbiota (202). However, these advanced techniques provide realistic and transformative tools for decoding MGBA mechanisms and identifying novel therapeutic targets for NDDs.

**FIGURE 2:**
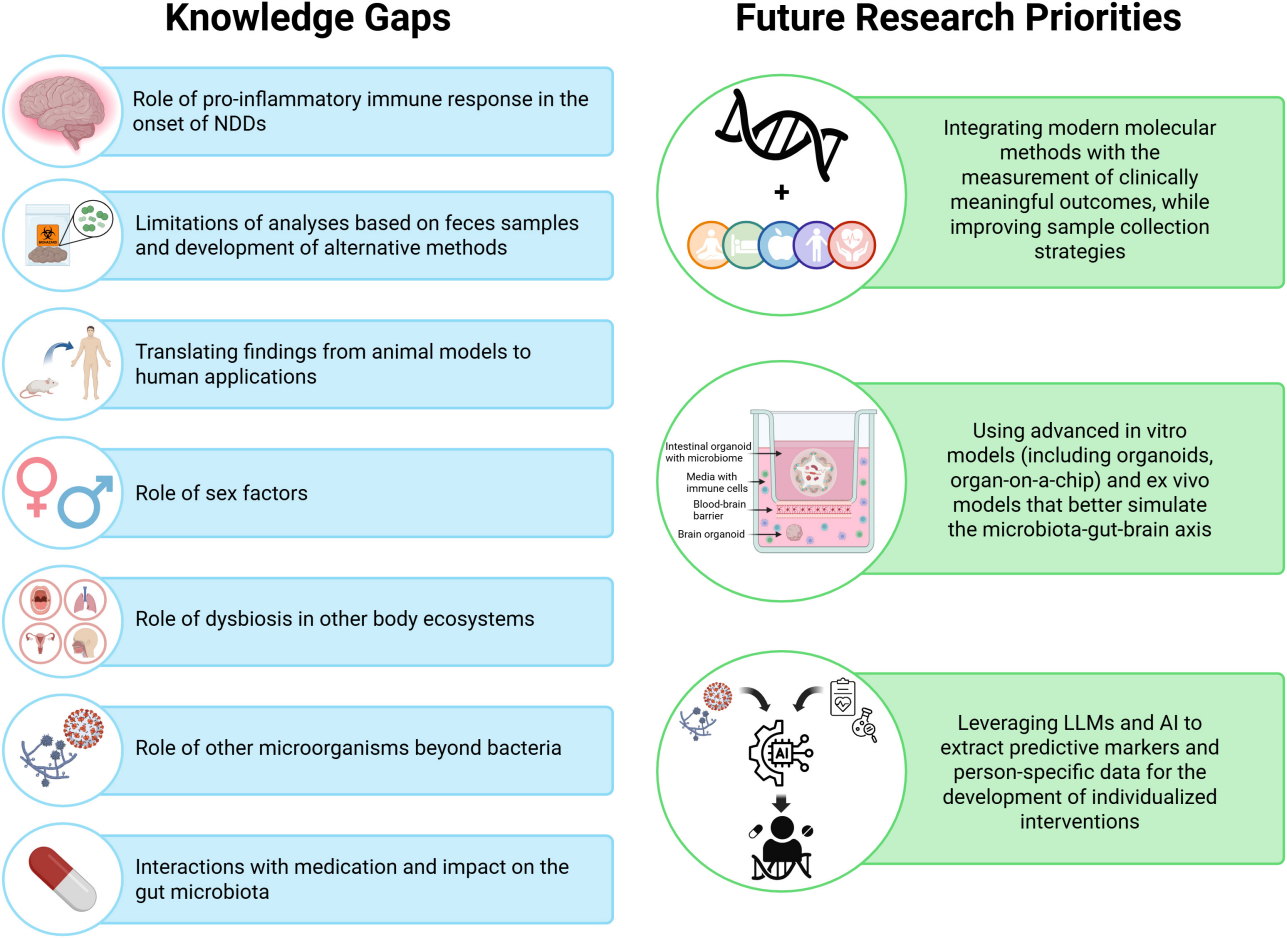
Schematic representation of knowledge gaps and future research priorities. Created with BioRender. AI, artificial intelligence; LLMs, large language models; NDDs, neurodegenerative diseases. Created with BioRender.com.

With outstanding advancements in artificial intelligence (AI), it is becoming increasingly clear that AI and machine learning (ML) will play an instrumental role in managing the complexity and dimensionality of microbiome data. These tools help extract robust, clinically relevant patterns from microbiome datasets which are massive and noisy in nature, to guide intervention and clinical trials design. For example, tools like SIAMCAT exemplify how carefully designed ML workflows can uncover disease-associated microbial signatures with predictive value beyond mere statistical associations (203). Furthermore, interpretable and rigorously validated ML models can offer a path toward reproducible biomarker discovery addressing the significant issue of inter-study variability (203). Notably, several open-access tools such as QIIME 2 (204) now incorporate ML functionalities, allowing for more accessible use of these algorithms in research. These advances could gradually cause a shift from exploratory correlation-based analyses to predictive modeling frameworks that prioritize generalizability and clinical relevance. In the context of NDDs, these AI-based models could help pinpoint microbiome-related diagnostic or prognostic indicators, or even stratify patients for tailored microbiome-based interventions. These tools could also help select the most appropriate strains or blends to use in a specific disease or to target specific metabolic pathways. Inter-individual variability in microbiota composition, host genetics, and environmental exposures means that a uniform intervention may not fit all (131, 205). Precision medicine, an emerging paradigm tailoring interventions based on individual characteristics, may provide maximum benefits while minimizing risks (86, 205). A summary of knowledge gaps and future research priorities is provided in Figure 2.

## 7 Conclusion

Recent developments in the understanding of the MGBA and of NDD pathophysiology support the continued investigation of microbiome-based supplement interventions for the prevention and clinical management of NDDs. Clinical studies show several positive benefits in the modulation of disease biomarkers and for the management of symptoms while having little to no side effects, but some clinical areas may have been overlooked such as mood and behavior changes in AD, and cognition in PD. These clinical areas present great potential for the development of new interventions. Likewise, clinical studies on ALS and HD are scarce, but preclinical evidence and growing knowledge of the MGBA and of NDD pathophysiology in general motivate the pursuit of clinical investigations. New technologies and methodological approaches offer great promise for the development and further refinement of microbiome-based interventions, which can not only provide substantial benefits, but also contribute to elucidating pathophysiology.
